# Alpha-Helical Protein Networks Are Self-Protective and Flaw-Tolerant

**DOI:** 10.1371/journal.pone.0006015

**Published:** 2009-06-23

**Authors:** Theodor Ackbarow, Dipanjan Sen, Christian Thaulow, Markus J. Buehler

**Affiliations:** 1 Laboratory for Atomistic and Molecular Mechanics, Department of Civil and Environmental Engineering, Massachusetts Institute of Technology, Cambridge, Massachusetts, United States of America; 2 Department of Materials Science and Engineering, Massachusetts Institute of Technology, Cambridge, Massachusetts, United States of America; 3 Center for Computational Engineering, Massachusetts Institute of Technology, Cambridge, Massachusetts, United States of America; Dalhousie University, Canada

## Abstract

Alpha-helix based protein networks as they appear in intermediate filaments in the cell’s cytoskeleton and the nuclear membrane robustly withstand large deformation of up to several hundred percent strain, despite the presence of structural imperfections or flaws. This performance is not achieved by most synthetic materials, which typically fail at much smaller deformation and show a great sensitivity to the existence of structural flaws. Here we report a series of molecular dynamics simulations with a simple coarse-grained multi-scale model of alpha-helical protein domains, explaining the structural and mechanistic basis for this observed behavior. We find that the characteristic properties of alpha-helix based protein networks are due to the particular nanomechanical properties of their protein constituents, enabling the formation of large dissipative yield regions around structural flaws, effectively protecting the protein network against catastrophic failure. We show that the key for these self protecting properties is a geometric transformation of the crack shape that significantly reduces the stress concentration at corners. Specifically, our analysis demonstrates that the failure strain of alpha-helix based protein networks is insensitive to the presence of structural flaws in the protein network, only marginally affecting their overall strength. Our findings may help to explain the ability of cells to undergo large deformation without catastrophic failure while providing significant mechanical resistance.

## Introduction

Catastrophic phenomena that afflict millions of lives, ranging from the failure of the Earth’s crust in earthquakes, to the collapse of buildings, to the failure of bones due to injuries, all have one common underlying theme: the breakdown of the basic constituents of any material ultimately leads to the failure of its overall structure and intended function. The failure and deformation of engineering materials has been studied extensively and has impacted our world by enabling the design of complex structures and advanced devices. However, the mechanisms of failure in biological systems are not well understood yet, thus presenting an opportunity to generate novel concepts to initiate a new paradigm of materials science. In order to provide a bottom-up description of materials behavior from a fundamental perspective, here we apply an atomistic multi-scale simulation approach that considers the structure-process-property paradigm of materials science and the architecture of proteins from the atomistic level up to the overall structure.

The cell’s cytoskeleton plays a crucial role in determining the overall cellular mechanical and biological properties. It consists of three major protein networks, actin, microtubules and intermediate filaments (IFs). Thereby, actin filaments and microtubules, both built from globular proteins, are responsible for cell dynamics and motility as well as particle transport [1]. However, these networks are rather “brittle” and break either at relatively low stress or low strains lower than 50% [2]. The third component of the cell’s cytoskeleton are alpha-helix based intermediate filament protein networks. In contrast to actin filaments and microtubules, intermediate filaments withstand much larger strains of up to several hundred percent [3], [4]. Thereby, they exhibit a highly nonlinear stress-strain relationship, being rather soft and mechanically “invisible” at small deformation, and become stiffer and more resistant against rupture at large deformation. This behavior is known as strain stiffening [5], [6]. Intermediate filaments also form the structural basis for lamin intermediate filaments, which constitute an important part of the cell’s nuclear membrane [7], [8], [9], [10], [11]. Similar to intermediate filaments in the cell’s cytoskeleton, lamin intermediate filaments fulfill the roles of defining the mechanical properties of the nuclear membrane and participate in gene regulation [7], [8], [9], [10], [11]. Their mechanical role has been demonstrated in several studies, which includes analyses of disease mechanisms in the rapid aging disease progeria [12].

**Figure 1:**
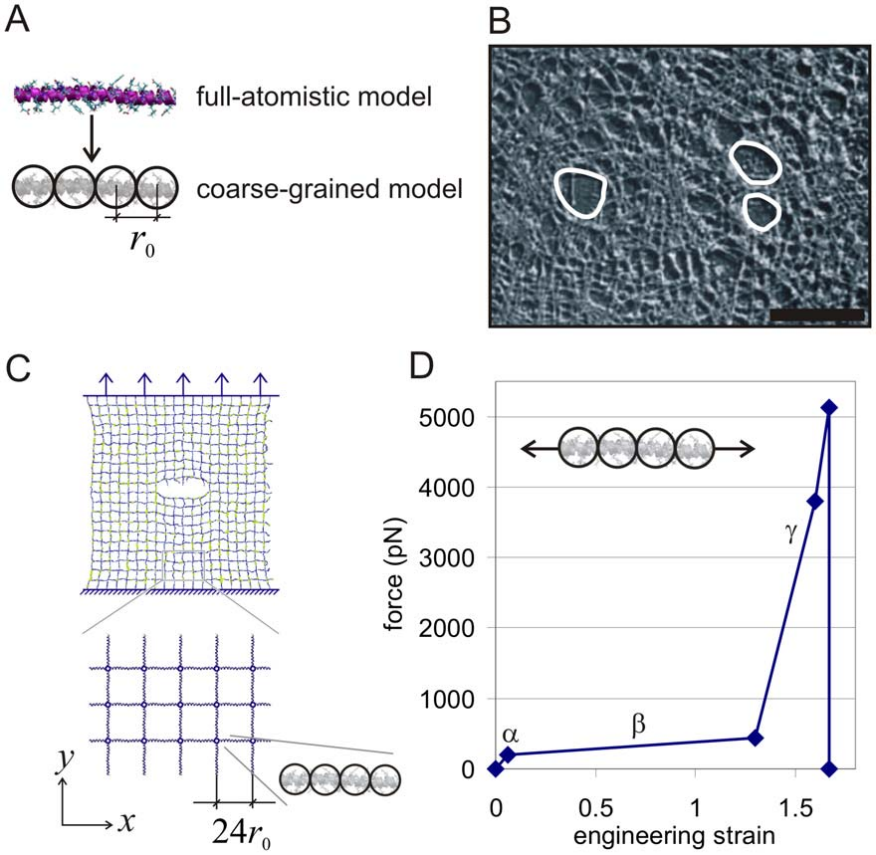
Model formulation, geometry and setup. Subplot A shows a schematic of the coarse-graining procedure, replacing a full atomistic representation of an alpha helical protein domain by a mesoscale bead model with bead distance *r*_0_. Subplot B shows a snapshot of a quasi-regular lamin meshwork (scale bar 1 µm) as observed in experimental imaging of oocytes; where structural imperfections are highlighted in white. Image of lamin meshwork reprinted with permission from Macmillan Publishers Ltd., from *Nature* [43], copyright © 1986. Subplot C depicts a schematic of the coarse grained protein network geometry used in this study, with the applied mode I tensile boundary conditions. The size of the network equals to 24 nm×24 nm, where each filament is represented by one alpha helix, as shown in the blow-up. A constant strain rate is applied in *y-*direction to apply mode I tensile loading through displacing the outermost rows of beads. The crack represents a geometrical flaw or inhomogeneity as they appear *in vivo*. Subplot D depicts characteristic force-strain curves for pulling individual alpha-helices as used in our mesoscale bead model. As explained in Materials and Methods, this force-strain behavior is derived from full-atomistic simulations and theoretical analysis, and has been validated against experimental studies. The labels α, β and γ identify the three major regimes of deformation.

**Figure 2:**
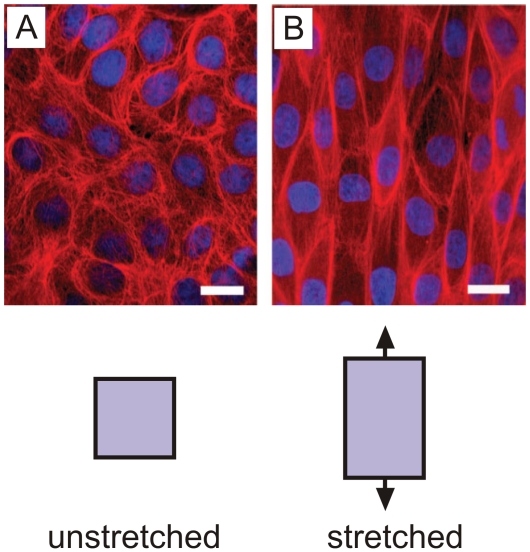
Effect of large uniaxial stretch on the intermediate filament network in MDCK cells, illustrating the ability of intermediate filament network to undergo very large deformation without catastrophic failure. The cells were grown on collagen-coated silastic membranes and stretched using a custom cell stretcher that was mounted on a confocal microscope. Cells were fixed and stained for immunofluorescence (red = keratin IFs, blue = DNA). Subplot A: Control cells were processed on a relaxed silastic membrane. Subplot B: Stretched cells were fixed, stained and imaged on membranes that were held in the stretched state. The approximate uniaxial strain in stretched cells is 75%. Scale bar is approximately 25 µm. Images reprinted with permission of John Wiley & Sons, Inc. from reference [46], *Biomechanical properties of intermediate filaments: from tissues to single filaments and back*, Vol. 29, No. 1, 2007, pp. 26–35, copyright © 2007 John Wiley & Sons, Inc.

Due to the superior mechanical response to large deformation and stress, it has been suggested in the biological literature that intermediate filaments play the role of cells’ “security belts” by providing structural support under rapid, large and severe deformation [4], [13]. The underlying protein motif that provides the constituents to build larger-scale networks of intermediate filaments (which appear at scales of tens to hundreds of nanometers) is the alpha-helical protein domain (see Figure 1A, top part). Extended alpha-helical protein domains assemble into larger-scale filaments that form mesh-like protein networks. A snapshot of the lamin intermediate filament network is shown in Figure 1B. It can be seen that whereas the network is rather regular in some regions, structural imperfections appear throughout. Figure 2 shows the effect of large uniaxial stretch on the intermediate filament network in Madin-Darby canine kidney (MDCK) cells, illustrating the ability of intermediate filament network to undergo very large deformation without catastrophic failure, where strain is distributed rather evenly throughout the tissue and consequently through the intermediate filament network.

**Figure 3:**
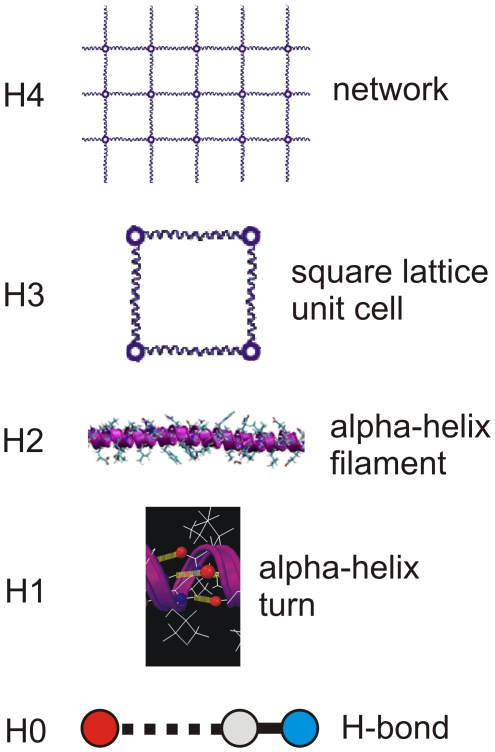
Hierarchical structure of the alpha-helical protein network considered here. The plot shows a schematic of five levels of hierarchies (H0..H4). Intrabackbone H-bonds provide the basic structural building block (H0). A cluster of 3–4 H-bonds stabilize the basic building block of alpha-helices, a alpha-helical convolution (H1). The linear arrangement of many convolutions leads to an alpha-helix filament (H2). The squared arrangement of several alpha-helix filament (H3) provides the basic structure of the network level (H4). The structure at the network level (H4) may also contain structural defects, as illustrated in Figure 1C.

The focus of this paper is on understanding the role of the alpha-helical protein motif under mechanical deformation of larger-scale protein networks, without and with structural (geometric) imperfections. To achieve this, we consider the deformation and rupture behavior of a simple model of an alpha-helix based protein network, as shown in Figure 1C. Figure 3 shows the multi-level hierarchical structure of the alpha-helical protein network considered here, involving five levels of hierarchies. The plot illustrates how individual H-bonded alpha-helical protein filaments are connected to form a macroscopic mesh structure. We emphasize that the goal of our model is not to accurately reflect a particular type of a protein structure. Rather, it is formulated deliberately as a general model to probe fundamental properties of a broader class of protein materials in which alpha-helix based protein filaments connect to form larger-scale networks. Despite its simplicity, our model captures the essential physical properties of individual alpha-helical protein filaments as identified in earlier theoretical and experimental studies. Through simulation of a larger-scale network, our model enables us to provide an important link between single molecule properties and mechanisms and the overall material behavior at much larger length-scales. (Details about the model formulation are included in the “Materials and Methods” section.)

In the literature, alpha-helical protein materials have been studied either from a macroscale perspective or from a single-molecule level, but not from an intermediate “mesoscale” viewpoint. For example, alpha-helix based intermediate filament networks have been investigated through shear experiments of protein gels [2] as well as through *in situ* studies with particle tracking rheology [14], where their material properties have been explored from a macroscopic perspective. On the other hand, the mechanical properties of the elementary nanoscale alpha-helical building blocks were studied extensively, and several publications have reported advances in the understanding of their nanomechanical behavior from both experimental [15], [16] and theoretical [17], [18], [19], [20], [21] perspectives.

Up until now the properties of alpha-helical protein networks specifically at the mesoscale have not yet been investigated, and no analysis of the rupture behavior of these networks was reported, despite their widely accepted significance of the mechanical performance and integrity. This has thus far hindered the formulation of bottom-up models that describe the structure-property relationships in protein networks under large deformation, which may explain their characteristic mechanical behavior. In particular, it remains unknown what the mechanism is by which these protein networks can sustain such large deformation of several hundred percent without catastrophic failure. This is an intriguing question since protein networks typically feature structural irregularities and flaws in their network makeup, as highlighted in Figure 1B. In synthetic materials (such as polymers, metals or ceramics), flaws typically lead to catastrophic failure at relatively small strains (often less than a few percent), preventing a material from undergoing very large deformation, reliably. This is because crack-like imperfections are generally responsible for initiating catastrophic failure [22], because they lead to very large stress concentrations at the corner of the cracks.

## Results and Discussion

**Figure 4:**
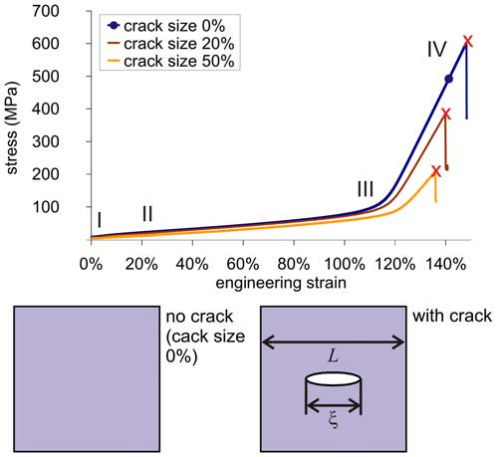
Mechanical response of the alpha-helical protein network. The graph shows stress-strain curves of a protein network, with and without a crack, as well as for two different crack sizes. The relative crack size is given as ratio of crack length *ξ* divided by the system size *L*, defined as *χ*  = *ξ* / *L*. We observe two major regimes, (I–III) a very flat increase in stress until approximately 100 MPa, followed (III–IV) by a very steep increase in stress due to strain hardening of the protein backbone up to strains of close to 140..150%. Eventually, strong bonds between different alpha-helical protein chains break, and the entire system fails catastrophically. Interestingly, there exists only little difference in terms of the failure strain between all three systems, indicating the fault tolerance of the studied structure. The perfect system (without a crack) has a strength of ≈600 MPa.

We begin our analysis with carrying a tensile deformation test of an alpha-helical protein network, by using the geometry and loading condition as shown in Figure 1C. We consider two geometric arrangements, as depicted in Figure 4 (lower part). First, a perfect protein network without a structural flaw. Second, a protein network with a structural flaw, here modeled as a crack-like inclusion. The goal of this analysis is to identify how an alpha-helical protein network responds to mechanical deformation under the presence of the crack.

We stretch both systems by displacing the outermost rows of the protein network and measure the stress-strain response of this material, until failure occurs. Figure 4 depicts stress-strain curves of the protein network with and without a crack, and for two different relative crack sizes (where the relative crack size is defined as ratio of crack length *ξ* divided by the system size in the *x*-direction *L*, defined as *χ*  = *ξ* /*L*). We consider a case *χ  = * 20% (the length of the crack is 20% of the size of the structure in the *x*-direction) and *χ  = * 50% (the crack reaches half way through the structure). The purpose of considering different crack sizes is to measure the effect of the size of the structural imperfection on the mechanical properties. For all cases considered we observe two major regimes in the stress-strain response, (I–III) a very flat increase in stress until approximately 100 MPa, followed (III–IV) by an increasingly steep increase of the stress, which lasts up to stresses close to 600 MPa (IV). Eventually, strong bonds between different alpha-helical protein chains break, and the entire system fails catastrophically. The increase of the stress in regimes (III–IV) is reminiscent of a phenomenon referred to as strain hardening. The systems with cracks fail at a slightly lower stress and lower strain than the perfect system. However, all three systems reach a remarkable strain to failure in excess of 135%. This means that the material can be extended by a factor of 2.35 times its initial length without breaking.

**Figure 5:**
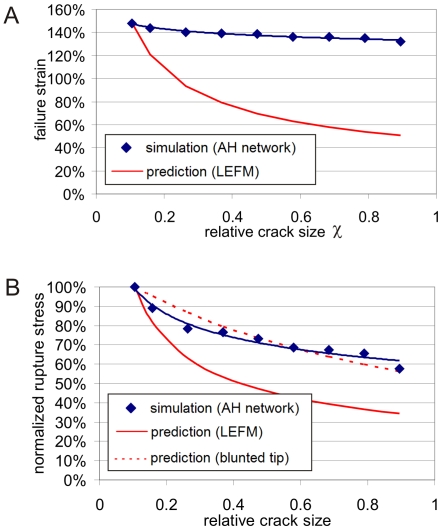
Failure strain and failure stress as a function of crack size and comparison with theoretical model. Panel A: Systematic analysis of the failure strain of the system, showing the failure strain over the relative crack size *χ*. The simulation results show that the failure strain is largely insensitive to the presence and size of cracks. Further, the plot includes the prediction based on eq. (2), corresponding to a scaling as 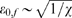. This behavior reflects that the scaling parameters are much different (−0.0362 vs. −0.5), and that linear elastic fracture mechanics (LEFM) fails to describe the fracture behavior of this material. Panel B: Analysis of the failure stress of the system as function of *χ.* The analysis also shows a deviation from the prediction of LEFM. The blunted crack-tip model is also shown for comparison (dashed line), providing an overall better fit than LEFM through the scaling law *σ*_0,*f*_ ∼ 1/(1+*Cχ*). Note that for relative crack sizes <5% the maximum strain and stress in panels A and B, respectively, does not change as the material has reached a complete insensitivity with respect to imperfections (data not shown in graph).

Figure 5A plots the failure strain as a function of the relative crack size, for a wide range of values of *χ* . Interestingly, the failure strain does not vary much among all systems, and even for a crack size of 80%, the material reaches a failure strain that exceeds 130%. This data shows that despite the presence of a flaw inside the protein network, the overall mechanical behavior remains intact and is not severely compromised by the structural imperfection. We find that the maximum stress depends more strongly on the size of the crack as shown in Figure 5B. However, even the system with 80% crack size still reaches 57% of the strength of a perfect structure without any defects. This performance is unmatched in most synthetic materials, where even small cracks can lead to a reduction of the strength by orders of magnitudes.

**Figure 6:**
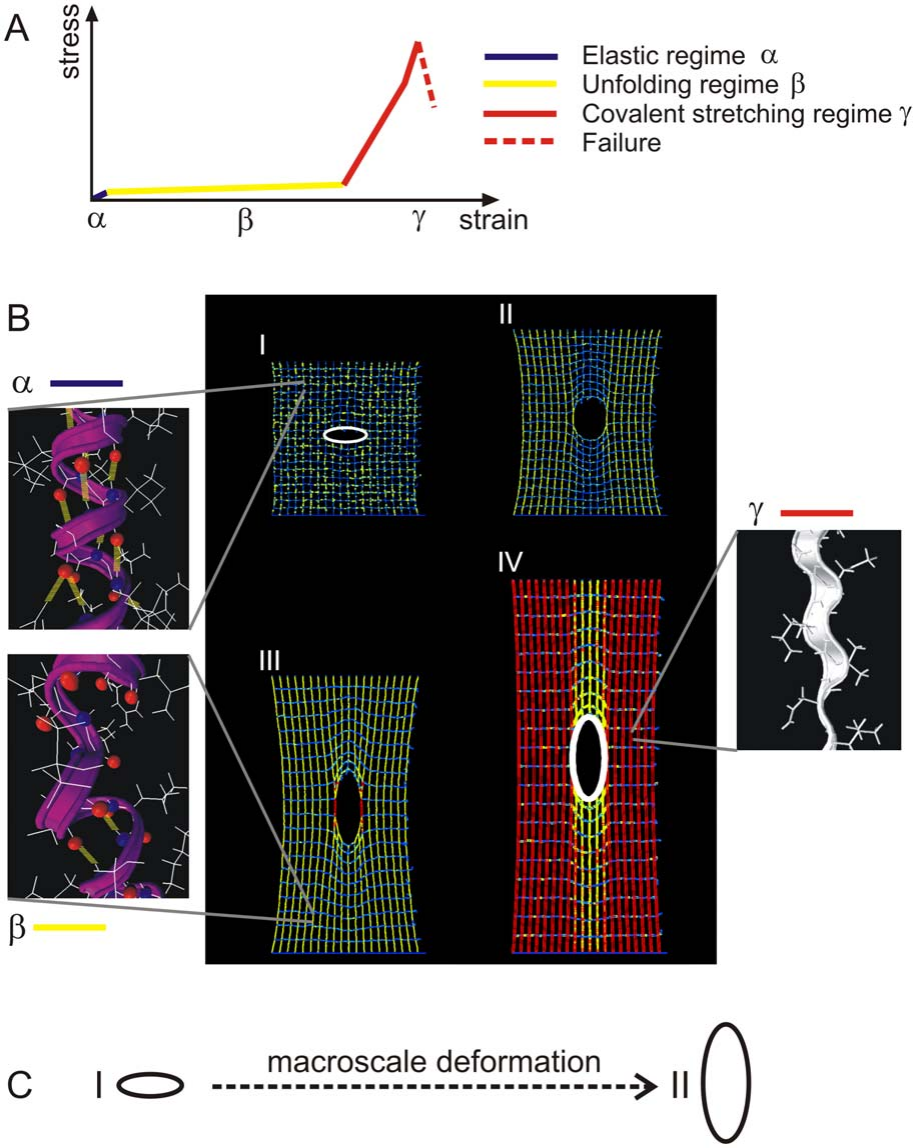
Snapshots of the protein network deformation. Panel A shows a schematic of the characteristic force-extension curve of a single alpha helix (consisting of three regimes) to provide the color code for the snapshots shown below. Panel B shows snapshots of the network with crack at different laterally applied strains (snapshot numbering refers to points shown in Figure 4). The deformation mechanism of the network is characterized by molecular unfolding of the alpha-helical protein domains, leading to the formation of very large plastic yield regions. These plastic yield regions represent an energy dissipation mechanism to resist catastrophic failure of the system. Once the entire structure reaches the rupture strain the crack propagates, leading to catastrophic failure, characterized by breaking of backbone atomic bonds as shown in the circled areas I and II. The white ellipsoids in the first and the last snapshot highlight the crack shape transformation that occurs during deformation (they show the surface geometry of the crack). The blowups show the nanoscale structural arrangements of the alpha-helical protein filaments under different levels of strain. The α structure is an intact helix, with 3–4 H-bonds per turn (yellowish thick lines). The β structure is a partially unfolded alpha-helix, with some of the H-bonds broken along the filament axis whereas others are still intact. The γ structure shows a completely unfolded alpha-helix, where the protein's backbone is being stretched. These three structures correspond to the color codes blue, yellow and red, respectively. Panel C shows the change of the crack geometry under macroscale deformation (crack shapes correspond to the white ellipses in panel B).

To explain this behavior, we carry out a detailed analysis of the deformation mechanism, as shown in Figure 6, where the color of the alpha-helical filaments indicates how much it has been deformed (specifically identifying: the elastic regime α – stretching of the alpha-helix without H-bond breaking; the plateau regime β – uncoiling of the alpha-helix through breaking of H-bonds; and the covalent stretching regime γ – the regime where the protein backbone is being stretched). We find that the deformation mechanism of the network is characterized by molecular unfolding of the alpha-helical protein domains, leading to the formation of very large yield regions (Figure 6B, snapshots II–IV; where the yield regions appear first in yellow and then in red color). These yield regions represent an energy dissipation mechanism to resist catastrophic failure of the system (we thus refer to them as “dissipative yield regions” in the following). Rather than dissipating mechanical energy by breaking of strong molecular bonds, the particular structure of alpha-helical proteins makes it possible that mechanical energy is dissipated via a benign and reversible mechanism, the breaking of H-bonds. Catastrophic failure of the structure does not occur until a very large region of the structure has been stretched so significantly that the strong bonds within and between alpha-helical protein filaments begin to fail. As shown in Figure 6B through the highlighted crack shape, we observe that the formation of yield regions enables a significant change of the shape of the crack, from an initial ellipsoidal shape where the longest axis points in the *x*-direction (Figure 6C, part I) to an ellipsoidal shape where the longest axis points in the *y*-direction (Figure 6C, part II). This microscopic change of the crack shape induced by the macroscopic applied load is an interesting cross-scale phenomenon with important implications on the failure behavior of the system, as will be discussed shortly.

**Figure 7 pone-0006015-g007:**
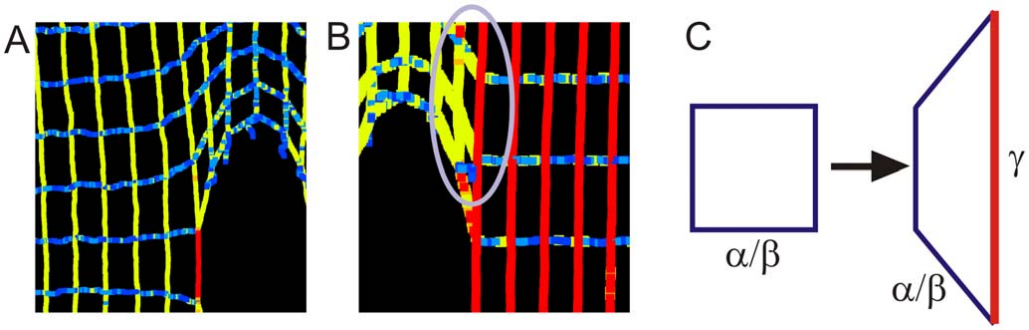
Detailed view into structure at crack tip for two distinct strain levels, and illustration of microscopic deformation mechanism. Panel A shows results associated with Figure 4, snapshot III. Panel B shows results associated with Figure 6, snapshot IV. The same color code as shown in Figure 6A applies here. The results depicted in panel A reveal that the filaments are relaxed in the *x*-direction (orthogonal to loading), and are highly stretched in the *y*-direction (direction of loading). There is a slight stress concentration at the tip of the crack, as can be seen by the red color indicating stretching of the protein filament's covalent backbone. In panel B, alpha-helices of the entire domain to the right (and left) of the crack are unfolded and the alpha helix protein backbones are stretched, whereas only the center part of the system has unfolded. This indicates that stress localization does not appear; instead, the entire network carries the load. This could explain the different behavior compared with conventional LEFM (see Figures 5). Panel C illustrates a possible microscopic deformation mechanism (as seen similarly in the circled area in panel B). The particular geometry of the square-lattice structure provides the structural basis for filaments to independently stretch without affecting neighboring bonds, since there are no immediate interactions between individual filaments in the network that prevent microscopic rotations and shear. This facilitates extremely large strain gradients at low energy cost (≈2x10^11^%/Å).

Figure 7 shows a detailed view into structure at crack tip for two different strain levels. Figure 7A shows results associated with Figure 6B, snapshot III. Figure 7B shows results associated with Figure 6B, snapshot IV. The same color code as shown in Figure 6A applies for the visualizations shown in Figure 7. The results shown in Figure 7A reveal that the filaments are relaxed in the *x*-direction (orthogonal to loading), and are highly stretched in the *y*-direction (the direction of loading). There is a slight stress concentration at the tip of the crack, as can be seen by the red color indicating stretching of the protein filament’s covalent backbone (whereas filaments in the immediate vicinity are strained less). In Figure 7B, the entire domain to the right (and left) of the crack has been unfolded and the backbone is stretched, whereas the center part of the system has just began to unfold.

In comparison with conventional materials, the protein based material considered here features intriguing fracture properties. To facilitate a systematic analysis we first calculate the fracture surface energy, an important quantity used to quantify the resistance of materials against failure [22]. With 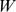 as the energy necessary to permanently break one alpha-helix (through rupture of strong backbone bonds), the fracture surface energy is defined as 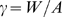, where *A* is the cross-sectional area associated with a single mesh element. Since *W*  =  1.63×10^−17^ J (obtained from the integral over the force-displacement curve of an alpha-helical element until breaking of the covalent backbone; it equals the area under the curve shown in Figure 1D), and *A*  =  1.2×10^−17^ m^2^ (length: 12×10^−9^ m, width: 10×10^−10^ m) the fracture surface energy is given by 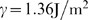. This is a value that is comparable to the fracture surface energy of silicon, which features 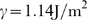 along the <111> crystal plane, albeit silicon has a much greater elastic modulus of *E*  =  243 GPa [23].

According to Griffith’s theory (also referred to as the “Linear Elastic Fracture Model”, LEFM) – a model often applied to describe fracture of conventional solids – the failure strain for a “central panel” through thickness crack inside a homogeneous material as the one considered here is given by
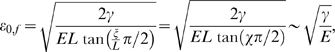
(1)where 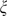 is the crack length, *L* is the system width in the *x*-direction, and *χ*  = *ξ* /*L* (see lower part of Figure 4 for the geometry and definition of variables). The scaling of 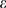 with respect to the elastic modulus 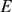 and the fracture surface energy 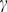 in eq. (1) partly explains the difference in failure strain observed in the alpha-helical protein network compared with materials such as silicon, which typically fail at less than a few percent strain. Due to the much lower modulus (approximately 3 GPa for alpha-helices in regime I–II, versus 243 GPa for silicon) but comparable fracture surface energy, the resulting failure strain is expected to be significantly enhanced in the protein material.

Furthermore, in conventional solids, the occurrence of singular stress concentrations is the reason for rapid catastrophic failure under deformation, as chemical bonds at the corners of cracks are stretched significantly and immensely exceed the deformation and stress imposed at the boundaries of the system. This type of behavior is not observed in the alpha-helical protein network. This is because each of the filaments is able to dissipate a significant amount of energy while they are able to independently stretch without affecting neighboring bonds, as illustrated in Figure 7C. This is possible since there are no immediate interactions between individual filaments in the network that prevent microscopic rotations and shear (aside from cross-links between filaments present at node points of the mesh). Therefore, these networks do not display a strong stress concentration at corners of cracks. In light of this observation, the relatively low density of protein filaments with open space between individual constituting elements, as well as their relatively small bending stiffness play a crucial role in defining the characteristic mechanical properties of the overall network.

In addition to the particular geometric arrangement in open networks, the properties of individual alpha-helical protein domains are decisive to explain this behavior. The high energy dissipation ability of individual alpha-helical protein filaments is achieved through the particular structure of alpha-helical proteins in combining a large array of small groups of H-bonds, which unfold concurrently in groups of 3–4 at relatively small force levels [21], [24], [25], providing a strongly nonlinear material behavior at the filament level as shown in Figure 1D. Notably, the utilization of H-bonds renders the structure self-healing, since H-bonds can reform at moderate temperature (e.g. body temperature) and thereby restore the initial alpha-helical structure even after severe deformation (provided that no strong bonds have been broken). In particular, since in the early relatively flat regime H-bonds are broken that can be reformed rather quickly, the formation of the yield zone that protects the integrity of the structure is effectively reversible upon relaxation of applied load at physiologically relevant time-scales.

We proceed with an analysis of the results in light of fracture models. Figure 5A displays an analysis of the failure strain of the system, plotting the failure strain over the relative crack size *χ* for both, the values measured from the simulation and the predictions from LEFM. The LEFM prediction for the scaling behavior of failure strain versus relative crack size is given by
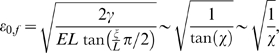
(2)suggesting a strong dependence of 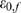 on 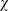. However, the simulation results clearly show that the failure strain is largely insensitive to the presence and the relative size of cracks. A power law fit of the form 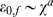 to the simulation data reveals that the failure strain 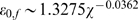. The prediction based on eq. (2) corresponds to a scaling as 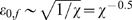. This analysis reveals that the scaling parameter *a* of 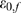 versus *χ* are much different (−0.0362 vs. −0.5), and that the conventional LEFM model fails to describe the failure behavior of this system. A similar analysis is shown in Figure 5B for the failure stress, comparing the prediction from LEFM to the measured dependence. Similarly as for the failure strain, the analysis shows that the failure stress remains significantly higher than the corresponding LEFM prediction even at very large relative crack sizes. However, the decay of failure stress is more rapid than the behavior found for the strain.

The behavior of the failure stress on the crack size is investigated further considering earlier solutions for cracks in elastomers [26], which have been developed specifically for the behavior of systems that show strong nonlinear (hyperelastic) and large-deformation elasticity. The maximum strength of the protein network (≈600 MPa) is about 11 times larger than the small-strain elastic modulus (≈56 MPa). This satisfies the criteria for elastic crack tip blunting as discussed in [26]. In agreement with the prediction put forth in [26], large blunting of the tip before failure is observed in the mesoscale experiments (see Figures 6 and 7). However, the model for fracture initiation for elastomers put forth in [26] is not directly applicable to our case, since the mechanisms such as void formation or microcracking are not observed in the alpha-helical protein network.

**Figure 8:**
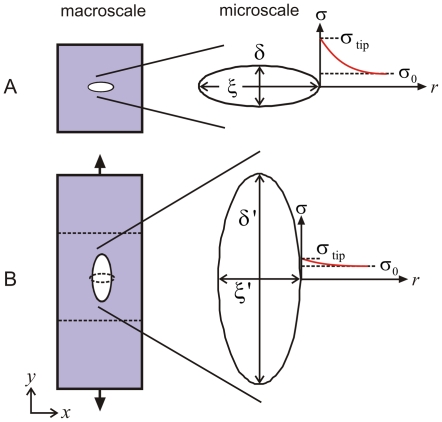
Change of the microscopic crack shape as the protein network undergoes macroscopic mode I tensile deformation. Panel A shows shape of the initial crack (an elliptical geometry where the length in the *x*-direction is much greater than the extension in the *y*-direction). Panel B shows shape of the final crack before onset of failure, representing an elliptical geometry where the length in the *y*-direction is much greater than the extension in the *x*-direction. The plots also indicate the distribution of stresses for both cases (the solution for the stress field is symmetric, but shown here only for the right half). The crack shapes reflect those measured in the simulations shown in Figure 6 (there highlighted in white color). The initial geometry and crack shape is shown in panel B (left part) in dashed lines to illustrate the significant transformation.

To overcome this limitation we present a simple analysis specific to our case, used here to develop a failure criterion for the alpha-helix protein network. The starting point is the observation that the crack shape significantly changes under the applied load and forms an elliptical geometry before the final stage of deformation associated with the higher stiffness, leading to an elliptical crack shape with a blunted crack tip (Figure 6C). A simple approximation of stress fields at a blunted crack tip can be obtained using the Inglis solution for elliptical cracks [27] (see schematic in Figure 8 with explanation of variables), where the crack tip stress is given by
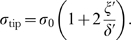
(3)


In eq. (3), 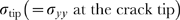 and 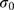 are the stresses at the tip and the far-field respectively, and 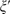 and 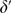 are the *x* and *y*-axes lengths of the elliptical crack shape before failure. Specifically, the parameters 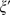 and 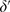 describe the transformed crack geometry after blunting has occurred through formation of large yield regions mediated by protein filament stretching, but before the final stage of deformation has begun (*i.e.*, before stage II–III shown in Figure 4). We note that the parameters 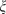 and 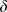 describe the initial crack geometry at the beginning of the simulation, before the transformation has occurred.

Equation (3) can be used to make a few interesting points. The equation provides a simple model for the reduction of stress magnification at corners due to structural transformation as discussed above. For an ellipsoidal crack shape where the longest axis points in the *x*-direction, the ratio 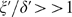 (Figure 6C, part I), the stress at the tip is much larger 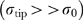 than for an ellipsoidal crack shape where the longest axis points in the *x*-direction, the ratio 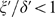 (Figure 6C, part II), where 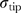 is only slightly larger than 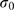. For example, for the geometry shown in Figure 6 the initial ratio 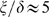, leading to 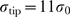. After the crack shape transformation has occurred, 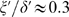, leading to 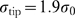, reduced by a factor 5.7.

We may also use eq. (3) to develop a simple model to predict the failure stress as a function of the crack size, accounting for crack blunting. The parameters 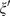
and 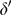 are related to the *x* and *y*-axes lengths of the undeformed initial elliptical crack 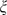 and 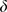. By assuming a first order linear relation 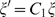 and 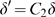 to describe the geometric transformation, we find that
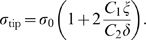
(4)We note that 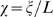 is used to express 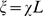, and therefore
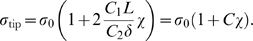
(5)The parameters 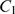
and
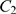
are generally functions of the applied strain. However, noting that failure strain is almost constant independent of crack size (see Figure 5A), we assume that 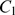 And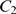 take the same respective value for different crack sizes at failure. It is noted that eq. (5) contains a constant prefactor 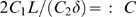. The crack will start to propagate when the condition 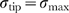 is satisfied, where 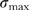 is the failure strength of a perfect alpha-helical network (since there are no other failure mechanisms such as void or microcrack formation [26], [28] present here). Combining these assumptions with eq. (5), we arrive at:
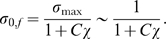
(6)


Equation (6) is a similar scaling law as proposed in eq. (2), but features an unknown parameter *C* that effectively describes the geometric change of the blunted crack tip under elastic deformation. This parameter can be identified by carrying out a least-squares curve fit for *C* to the range of geometries considered in our computational experiments, leading to *C* = 1.102. The results are shown in Figure 5B, revealing a much better agreement with the simulation data; albeit the model itself is empirical due to the existence of a fitting parameter that must be determined from experimental measurements. However, the model is useful as a constitutive equation to predict the strength of alpha-helical protein networks that can be used in larger-scale simulation methods (e.g. finite element models) to describe the strength behavior of such materials. It might also be used as a design tool to construct systems with optimized values of *C* that provide less sensitivity to the crack size *χ* (where possible changes to the geometries at different hierarchical levels as shown in Figure 3 could be used as design variables). Possible improvements of the model might be obtained using quantized fracture mechanics models [29] or the development of formulations that account for the specific elastic properties of the system considered here.

It is noted that the definition of “failure” as considered here involves breaking of strong backbone bonds in the network. Under typical physiological conditions this may not occur, since deformation is largely limited to reversible processes at smaller stresses. However, the analysis put forth here provides a worst case scenario to identify the limit of mechanical deformation, which shows that even at modest stresses extremely large deformation can be accommodated without causing any harm to the network integrity. Further, failure modes that may be observed in other systems entail intermolecular sliding of filaments [30], [31], [32]. The analysis discussed here still holds; with the distinction that sliding prevents immediate catastrophic failure of the system but instead leads to the formation of a “plastic zone”, formed by the domain in which filaments have begun sliding. This plastic zone provides further resistance against catastrophic breakdown. Indeed, sliding mechanisms have been suggested for intermediate filament protein structures [30], [31], [32].

### Conclusion

The main result put forth in this paper is that it is due to the particular structure and properties of alpha-helical protein constituents that enable the formation of large dissipative yield regions and a severe structural transformation of the crack shape, which effectively protects alpha-helical protein network against catastrophic failure (Figure 6). These yield regions provide a means to dissipate mechanical energy before strong bonds are being stretched and broken, and enables the system to undergo deformation well beyond 130% strain even when cracks are present that stretch of up to 80% of the system size. As a result of formation of dissipative yield regions, the alpha-helical protein networks are largely insensitive to structural flaws, which is reflected in the diminutive influence of the crack size on the failure strain (Figure 5A) and the failure stress (Figure 5B). This behavior is referred to as flaw tolerance.

The comparison with the scaling behavior predicted from conventional fracture models as summarized in Figure 5, and the characteristic failure mechanisms highlighted in Figures 6–8 illustrates the distinct behavior of alpha-helical protein materials. Table 1 provides a summary of the roles and mechanisms of individual levels of structural hierarchies shown in Figure 3 for the overall system behavior, illustrating that each hierarchical level plays a key role in achieving the overall system performance. The dominating unit deformation mechanism f alpha-helical protein networks is protein unfolding mediated by continuous rupture of clusters of H-bonds, as shown in Figures 6. The detailed fracture mechanism is summarized as follows:

- Initially, the system is loaded in mode I (tensile load), with the load applied vertically to the long axis of the crack. In solids, this represents the most critical mode of loading with respect to inducing high local stresses in the vicinity of the crack tip.
- As load is applied, the protein filaments start to unfold, as H-bonds begin to rupture and the alpha-helical proteins uncoil (see blowups shown in Figure 6B).
- The system elongates in the loading direction, and the shape (morphology) of the crack undergoes a dramatic transformation from mode I, to a circular hole, to finally an elongated crack aligned with the direction of loading (see schematic depicted in Figure 6C and Figure 8). This transformation is caused by the continuous unfolding of the individual proteins around the crack, which can proceed largely independently from their neighbors.
- As discussed in the crack blunting model shown in Figure 8, the elongated crack features rather small stresses in the vicinity of the crack. The transformation of the crack shape is thus reminiscent of an intrinsic ability of this material to provide self-protection.
- The almost identical strain at fracture (Figure 5A) is due to the similar stretching mechanism and unfolding of the proteins at the initial stages of loading. Due to the self-protection mechanism and the related change of the crack shape (that is, the alignment along the stress direction) the crack becomes almost invisible, even if dominating large parts of the cross-sectional area, and has little adverse effect on the overall system performance.

**Table 1:** Role and mechanism of individual levels of structural hierarchies for overall system behavior, illustrating that each hierarchical level plays a key role in achieving the overall system performance.

Hierarchy level H*n*	Description	Key mechanism(s)
H0	Level of chemistry; Intrabackbone H-bond; Basic chemical bonding, enabled by particular polypeptide structure	H-bonds form at moderate temperatures; Drive self-assembly of alpha-helices
H1	Alpha-helix turn defined by cluster of 3–4 H-bonds; Basic building block of alpha-helix filament	Clusters of 3–4 H-bonds provide optimal resistance against mechanical failure [25] (3–4 H-bonds break concurrently, providing maximum possible mechanical strength at minimal material cost)
H2	Alpha-helix filament; Basic building block of square lattice	Particular geometry with linear array of turns provides structural basis for large extensibility of >150% strain via repeated rupture of turns (see Figure 1D)
H3	Square lattice unit cell; Microstructural geometry of network level	Distance between filaments provides structural basis to independently stretch without affecting neighboring bonds, since there are no immediate interactions between individual filaments in the network that prevent microscopic rotations and shear (see Figure 7C); Facilitates extreme strain gradients at low energy cost (≈2×10^11^ %/Å)
H4	Network; Macroscopic functional scale (e.g. nuclear envelope for mechanical integrity)	Structural transformation of crack-like defects (see Figure 6C and 8) to mitigate stress concentrations

To the best of our knowledge, the studies reported here for alpha-helical protein networks are the first of its kind, providing insight into the fundamental deformation and failure mechanisms of an abundant class of biological materials that feature networks of similar protein filaments. Our results may further explain the ability of cells to undergo very large deformation (see, e.g. Figure 2) despite irregularities in the structural makeup of the protein network. This represents an intriguing ability of this class of materials to self-protect themselves against adverse effects of structural irregularities. Avoiding such structural irregularities in the material makeup would require a high energetic cost (e.g. through the need for strong bonding as it appears in crystalline solids). Biological materials solve this challenge by adapting a structure that is intrinsically capable of mitigating structural irregularities or flaws while maintaining high performance, representing a built-in capability to tolerate defects. These properties effectively result in self-protecting and flaw-tolerant materials.

Further investigation could be carried out to provide a more realistic description of the protein network. Our approach does not precisely reflect the specific nanostructure in lamin intermediate filaments as it was designed to provide a rather simple, generic description (see discussion above). Our assumption of a square lattice network of alpha-helical proteins does not accurately reflect the structure of many biological materials, and future investigations could be focused on describing the effects of the differences due to different nanostructural geometries. In these cases, additional levels of hierarchies would enter the structure shown in Figure 3, resulting in additional mechanisms of deformation and failure beyond those listed in Table 1. For example, sliding between alpha-helical constituents (e.g. in tetramers or larger-scale protein assemblies) could be an important failure mechanism, which would prevent the immediate drop of the stress to zero as assumed here once this failure mode begins to operate. The possibility of sliding as a deformation mode might explain the slightly lower maximum stress and a deviation from continuous stiffening as seen in experimental analysis of intermediate filament nentworks [32] (despite an overall agreement the stress-strain curve shape between experiment and simulation results; where there is a deviation at large stresses). A detailed analysis of the network in dependence of these effects, as well as a quantitative comparison is left to future studies. However, it is pointed out that the mechanisms of self-protection and flaw-tolerance as observed here still hold, because the basic characteristics of the protein network makeup remains similar. The focus on a simple model system as reported here - in the spirit of a model material [33], [34] - provides a clean and well-defined approach to elucidate fundamental mechanisms of failure initiation. If we had focused on attempting to model the particularities of a specific material we would not have been able to identify generic failure mechanisms.

Studies of the mechanical performance of alpha-helical based protein networks as reported here are crucial for advancing our understanding about the deformability, strength and failure behavior of protein materials in general, as well as for our ability to create *de novo* synthetic nanomaterials for application in biotechnology and synthetic biology. We speculate that our results may also explain the mechanical properties of other biopolymers such as spider silk, where analogous dissipation mechanisms might contribute to these materials' extreme strength and robustness against large deformation. Future studies will be necessary to explore effects specific to these materials. Our findings are also reminiscent of the sacrificial bond concept discussed earlier in the context of bone and other biopolymers [35], [36], [37], [38], albeit the sacrificial bond model has not yet been explored in the context of crack-like imperfections and its impact on mechanical performance. Earlier studies of the mineral crystal phase in bone have also pointed out flaw-tolerant behavior, which was linked to nanoscale confinement of mineral platelets [39].

In summary, our analysis, together with earlier studies of single molecule behavior of alpha-helical proteins, improves our understanding of deformation and failure mechanisms of structurally flawed protein networks by providing an integrative model to bring together single molecule properties and larger-scale material behavior through an integrated, consistent multi-scale perspective. A computational approach as put forth here is a promising method that complements experimental investigations. It can also be used to enable a systematic design of materials, by systematically expanding the structural levels shown in Figure 3 and by designing novel mechanisms beyond those listed in Table 1. This may one day provide a computational engineering approach similar to what is used in the design of cars, buildings and machines today, applied to the integrated approach that bridges multiple material levels in the design of materials and structures.

The field of genomics is concerned with the study of genes and their effects on macroscopic functions, and has led to considerable medical advances. Genomics, however, does not elucidate material properties, nor the mechanistic relation of hierarchical multi-scale structures and their resulting properties. The multi-scale behavior of protein assemblies with the goal of elucidating the relation between structure and material properties represents a grand challenge at the interface of materials science and biology. This gap in understanding can be closed by systematically studying the material properties of hierarchical protein structures and their effect on the macroscopic properties, an approach part of a larger effort to study the role of materials in biology, referred to as materiomics [40]. Here we have focused on the properties of alpha-helical protein networks by screening their mechanical performance under variations of their structural makeup across multiple hierarchical levels.

## Materials and Methods

### Model formulation

The basis for the network model is a coarse-grained description of an alpha-helical protein structure, referred to as a mesoscale bead model. In our model, the entire sequence of amino acids that makes up the alpha-helix structure is replaced by a collection of beads (see schematic in Figure 1A), where each bead represents hundreds of atoms in explicit solvent. This approach is adapted since it significantly reduces the computational cost of simulating a large protein network, enabling us to describe a large lattice-like network of strongly bonded alpha-helices (Figure 1B) (these bonds may be formed through intermolecular cross-links or strong electrostatic bonding). The beads in the mesoscale model interact according to an intermolecular multi-body potential, developed to reflect the key physical properties of individual alpha-helical protein domains including adhesion, stretching and bending. The total energy of the system is given by 
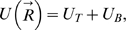
(6)where 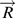 denotes the positions of all particles. The total energy is given by the sum over all pair-wise (that is, *U_T_*) and all three-body interactions (that is, *U_B_*), where 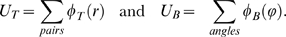
(7)Specific interparticle potential energy expressions are defined for each of the contributions given in eq. (4). We approximate the nonlinear force-extension behavior of alpha-helical proteins under tension by a multi-linear model. This multi-linear model is a combination of four spring constants 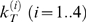 which are turned on at specific values of molecular stretch. A similar model has been used successfully in earlier studies of fracture in crystalline model materials [33], [34] and provides an effective way to describe the nonlinear constitutive behavior based on computationally effective, simple piecewise harmonic potential functions. Based on this model, the tensile force between two bead particles is described as:
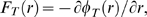
(8)(the energy function 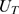 is given by integrating the force 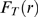 over the radial distance), where 
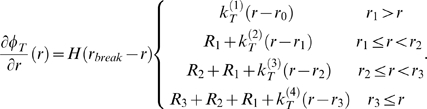
(9)In eq. (6), 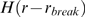 is the Heaviside function 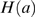 which is defined to be zero for 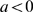, and one for 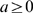. The parameters 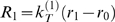, 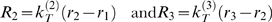 are calculated from force continuity conditions. The bending energy of a triplet of three bead particles is given by 
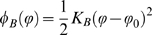
(10)with 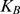 relating to the bending stiffness of the molecule 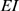 through 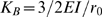.

### Model parameter identification

All parameters in the mesoscale bead model are determined from full atomistic simulation results and theoretical studies, based on careful studies reported in earlier publications that involve experimental validation [17], [18], [41]. We choose *r*_0_ = 0.5 nm per bead, providing significant computational speedup while maintaining a sufficiently fine discretization of the alpha-helical protein (leading to a bead particle mass *m* = 400 amu). All parameters in eq. (6) are fitted to reproduce the nanomechanical behavior obtained using the full atomistic model with the molecular formulation [18]. In particular, the stiffness in regime α (in Figure 1D) 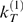 is identified from these simulations [18]. Further, a detailed analysis of the alpha helix behavior in dependence of the deformation rate was carried out in previous studies, where it was shown that for vanishing pulling rates the force at end of the first (see regime α in Figure 1D) and the beginning of the second regime (see regime β in Figure 1D) reaches an asymptotic value of ≈200 pN [21]. It was also shown that this value agrees with experimental measurements (as discussed in [21]) and we thus consider this in the formulation of our bead model to mimic quasi static deformation at vanishing pulling rates as relevant for physiological and experimental deformation speeds. This enables us to identify the onset point for the second regime, *r*_1_. The stiffness in regime β 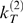 is identified from atomistic simulations [18]. The onset of regime γ in Figure 1D, described by parameter *r*_2_. is identified from atomistic simulation as well [41], which includes specifically the extraction of the transition strain and the stiffness parameters in regime γ (that is, 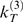 and 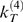 as well as *r*_3_). Bond rupture of the protein polypeptide backbone is modeled at forces of ≈5,500 pN, which provides the value for *r*_break_. This is based on earlier ReaxFF reactive force field results [41] (here we use a slightly smaller value for the rupture force than reported in [41] to reflect the behavior at vanishing pulling rates). The bond strength of several nN for strong bonds as used here is a value widely accepted in the literature and has also been measured experimentally [42]. Figure 1D depicts the force-strain curve for alpha-helices as reproduced by the mesoscale bead model. The bending stiffness is obtained from bending deformation calculations of alpha-helical molecules, as described in earlier publications [17], [41] (values are validated by comparison with the experimentally measured persistence length on the order of a few nanometers). The time step is chosen to be 15 fs. The entire set of parameters of the mesoscale model is summarized in Table 2.

**Table 2:** Summary of the parameters used in the mesoscopic molecular model, chosen based on full atomistic modeling of alpha-helical molecules (note that 1 kcal/mol/Å = 69.479 pN).

Parameter and units	Numerical value
Equilibrium bead distance 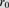 (in Å)	5.00
Critical distances 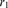 , 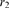 and 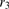 (in Å)	5.30, 11.50, 13.0
Tensile stiffness parameters 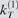 , 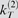 , 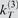 , 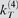 (all in kcal/mol/Å^2^)	9.70, 0.56, 32.20, 54.60
Bond breaking distance 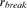 (in Å)	13.35
Equilibrium angle 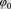 (in degrees)	180.00
Bending stiffness parameter 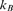 (in kcal/mol/rad^2^)	3.44
Mass of each mesoscale particle (in amu)	400.0

### System definition, geometry and boundary conditions

We create a network with a mesh side length of 12 nm (in square shape), which equals to 24 beads since *r*_0_ = 0.5 nm per bead (see Figure 1A–B). The linkers between the perpendicular filaments are modeled as beads that are freely deformable in both directions without any angular restraints. This mimics the existence of cross-links between individual alpha-helical filaments (facilitated e.g. through side-chain mediated bonds, such as disulfide bonding). As shown in Figure 1C, we create a square meshed protein network out of individual filaments, where each filament consists of a single alpha helix. The orthogonal arrangement of protein filaments roughly mimics an intermediate filament protein network as for example observed in lamins in the nuclear membrane of oocytes (see Figure 1B) [43]. We note that the choice of a single alpha helix per filament represents a limitation compared with the actual structure of intermediate filaments *in vivo*, which typically contains multiple alpha-helices arranged in parallel. However, the purpose of the present study is not to exactly model the structure of lamin, but rather provide a generic study on the behavior of alpha helical protein networks without and with defects. We deliberately avoid the attempt to model a specific protein filament. We consider a system with 20 filaments (each composed of 24 beads as discussed above); with an overall network size of 24 nm×24 nm. Pulling is applied in *y*-direction in mode I tensile loading, as indicated in Figure 1B. Thereby the first two rows of beads at the bottom are fixed. Displacement boundary conditions are applied to the upper three rows of beads, so that the upper three rows of beads are moved continuously following a prescribed strain rate. A strain rate of 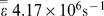 is used for all studies (studies with varying strain rates were carried out and it was confirmed that the system undergoes deformation near equilibrium at the strain rate chosen). All simulations are carried out at 300 K in a *NVT* ensemble (constant temperature, constant volume, and constant number of particles). The overall length scales reached in this study (several tens of nanometers) shows a sufficiently high level of repetition of individual meshes so that boundary effects can be neglected. Larger systems do not change the overall behavior described in this paper.

### Crack modeling

To model the crack-like inclusion, protein filaments across the crack surface are not connected from the beginning of the simulation (and can not reform). This approach effectively models the existence of a structural imperfection in the protein network through the existence of an elliptical flaw. By controlling how many protein filaments are broken at the beginning of the simulation we control the size of the crack.

### Stress and strain calculation

For calculation of stress the virial stress approach was applied [44]. The failure stress is measured at the point when filaments begin to fail (usually identified through a rapid drop of the stress). The failure stress data shown in Figure 4 is obtained through an average over the entire simulation domain. The stress at failure shown in Figure 5B is defined as the remotely applied stress; which is different than the measured average stress shown in Figure 4. It is calculated by taking the applied strain (due to a particular prescribed displacement) and computing the associated stress following the stress-strain response of a perfect crack-free system (see Figure 4, curve marked with •). The strain is defined by 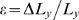 ( = engineering strain), where 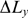 is the applied displacement and 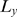 is the length of the system in the *y*-direction (the pulling direction).

### Simulation implementation

The mesoscale simulations following an MD scheme are implemented in the simulation package LAMMPS (Large-scale Atomic/Molecular Massively Parallel Simulator) [45]. For visualization we use the OpenDX package. The simulation model implementation in LAMMPS is available upon request. All simulations have been carried out at MIT's Laboratory for Atomistic and Molecular Mechanics on a Dell linux computing cluster with Intel Xeon dual core CPUs. One simulation takes approximately 24 hours to complete.
